# Interleukin-1 Inhibitors and Vaccination Including COVID-19 in Inflammatory Rheumatic Diseases: A Nonsystematic Review

**DOI:** 10.3389/fimmu.2021.734279

**Published:** 2022-01-27

**Authors:** Pamir Atagündüz, Gökhan Keser, Mehmet Soy

**Affiliations:** ^1^ Department of Rheumatology, School of Medicine, Marmara University, Istanbul, Turkey; ^2^ Department of Rheumatology, Faculty of Medicine, Ege University, Izmir, Turkey; ^3^ Department of Internal Medicine and Rheumatology, Faculty of Medicine, Altınbaş University, Istanbul, Turkey

**Keywords:** IL-1, interleukin-1 inhibitors, autoimmune/autoinflammatory rheumatic diseases, COVID-19, vaccination

## Abstract

Newly emerging variants of coronavirus 2 (SARS-CoV-2) raise concerns about the spread of the disease, and with the rising case numbers, the Coronavirus disease 2019 (COVID-19) remains a challenging medical emergency towards the end of the year 2021. Swiftly developed novel vaccines aid in the prevention of the spread, and it seems that a specific cure will not be at hand soon. The prognosis of COVID-19 in patients with autoimmune/autoinflammatory rheumatic diseases (AIIRD) is more severe when compared to the otherwise healthy population, and vaccination is essential. Evidence for both the efficacy and safety of COVID-19 vaccination in AIIRD under immunosuppression is accumulating, but the effect of Interleukin-1 on vaccination in general and in AIIRD patients is rarely addressed in the current literature. In light of the current literature, it seems that the level of agreement on the timing of COVID-19 vaccination is moderate in patients using IL-1 blockers, and expert opinions may vary. Generally, it may be recommended that patients under IL-1 blockade can be vaccinated without interrupting the anti-cytokine therapy, especially in patients with ongoing high disease activity to avoid disease relapses. However, in selected cases, after balancing for disease activity and risk of relapses, vaccination may be given seven days after the drug levels have returned to baseline, especially for IL-1 blocking agents with long half-lives such as canakinumab and rilonacept. This may help to ensure an ideal vaccine response in the face of the possibility that AIIRD patients may develop a more pronounced and severe COVID-19 disease course.

## 1 Introduction

With its newly emerging variants, severe acute respiratory syndrome coronavirus 2 (SARS-CoV-2) remains a challenging medical emergency. With the spread of the disease to every continent, case numbers continue to rise. As of the writing of this review, there were over than 226,8 million reported Coronavirus disease 2019 (COVID-19) cases and 4,6 million deaths, since China reported the first cases to the World Health Organization (WHO) in December 2019 (1).

COVID-19 has a poor outcome in hospitalized patients. The main cause of death in these patients is acute respiratory distress syndrome which is lethal in nearly 40% of hospitalized patients (2). Vaccination is the sole effective approach in ending the spread of the pandemic and preventing losses due to COVID-19 without a cure at hand. Pfizer/BioNTech (mRNA), Moderna (mRNA), AstraZeneca (adenovirus vector), Janssen (adenovirus vector), Sinopharm’s Covilo (inactivated SARS-CoV-2 virus), and Sinovac’s CoronaVac (inactivated SARS-CoV-2 virus) are approved by the WHO for global use (3–8). Sputnik V and Covaxin have been authorized for regional use in Russia and India, respectively (9, 10).

The protection after vaccination is reported to start after about 14 days, but even after full vaccination, breakthrough infections have been reported (11, 12). Even a single vaccination with mRNA vaccines provides about 70% protection against symptomatic disease, an 80% decreased hospital admission rate, and an about 85% dropped mortality rate (13).

Effective vaccination requires an intact immune system, and the degree of immunosuppression established in the treatment of autoimmune/autoinflammatory rheumatic diseases (AIIRD) with antirheumatic drugs including Interleukin-1 (IL-1) inhibitors, may compromise the immunization process (13–17). IL-1 inhibitors are widely used in the treatment of polygenic autoinflammatory diseases and monogenic periodic fever syndromes, most often as a lifelong treatment (18). Currently, how IL-1 blockade affects vaccination is scarcely investigated and the level of consensus and/or agreement on how to use and when to vaccinate for COVID-19 under IL-1 blockade is moderate (19).

We aimed to summarize the current literature on all forms of vaccinations under IL-1 blockade to extrapolate relevant data for the use and timing of COVID-19 vaccinations for patients receiving anti-IL-1 therapy.

### 2 Methods and Data Collection

To review vaccination under IL-1 blockade we did a Pubmed search with the following keywords from inception until June 2021, and reached a total of 1,280 peer-reviewed manuscripts published in the English language:

−IL-1 and autoimmune rheumatic diseases: Total = 544 (three meta-analyses, four systematic reviews, 209 reviews, three randomized controlled trials, 306 research articles, 13 case reports, and six editorials)
−IL-1 and autoinflammatory rheumatic diseases: Total = 155 (two systematic reviews, 71 reviews, one randomized controlled trial, 67 research articles, 13 case reports, and one editorial)
−IL-1 blockade and immunization: Total = 540 (one meta-analysis, three systematic reviews, 116 reviews, 398 research articles, nine randomized controlled trials, nine case reports, and four editorials)
−IL-1 blockade and vaccination: Total = 30 (six reviews, 24 research articles)
−Vaccination, IL-1 autoimmune rheumatic disease: Total = 9 (six reviews, two research articles, and one editorial)
−vaccination IL-1 autoinflammatory rheumatic disease: Total = 2 (one review and one research article = 1)

Meta-analyses, systematic reviews, reviews, randomized and non-randomized clinical trials, and case reports were used for the present review. After reading the title and/or abstracts 62 relevant articles addressing IL-1 biology and functions of IL-1 family cytokines, vaccination under IL-blockade in general, and vaccination for COVID-19 in patients with AIIRD under IL-blockade were selected.

## 3 Results

### 3.1 Biology of Interleukin-1

#### 3.1.1 Interleukin-1 Superfamily

Paracrine and autocrine functions of interleukins regulate the growth, differentiation, and activation of immune cells in inflammation and immune responses. Structurally, interleukins are proteins.

The IL-1 superfamily consists of 11 cytokines, IL-1F1 to IL-1F11. The family name IL-1F1 refers to IL-1α, IL-1F2 to IL-1β, and IL-1F3 to IL-1Ra. The two forms of IL-1, IL-1α and IL-1β, are the founding members of this family (20). IL-1α and IL-1β display a low level of amino acid sequence homology (27%), but with a similar three-dimensional structure, they bind to the same receptor, the type I IL-1 receptor (IL-1RI), for downstream signaling. IL-1R1 is found on almost all cells. A third naturally occurring ligand of this receptor, the Interleukin 1 receptor antagonist (IL-1Ra), inhibits signaling by competing with IL-1α and IL-1β (18). The type 2 IL-1 receptor (IL-1R2) functions as a decoy receptor for IL-1β and possibly inhibits the signaling (21).

#### 3.1.2 IL-1 Synthesis

When first discovered in 1943, this leukocyte released endogenous pyrogen was first termed as “pyrexin”, and it was the first identified interleukin Accumulating evidence suggests that the cytokines of the IL-1 family are the main mediators of innate immunity. Additionally, the differentiation and functioning of polarized innate and adaptive cells are orchestrated by the IL-1 family (22).

A well-functioning IL-1 superfamily is essential for the survival of the host but in a state of dysregulation or excessive activation, mainly autoinflammatory and sometimes autoimmune diseases may occur (20).

#### 3.1.3 Tissue Distribution and Secretion of IL-1

IL-1 has a wide distribution of expression. IL-1α is constitutively present intracellularly in epithelial and mesenchymal cell types and is not actively secreted, whereas IL-1β is specifically released under disease conditions. IL-1α and IL-1β are mainly expressed in macrophages of both lymphoid and non-lymphoid organs. Neutrophils, epithelial and endothelial cells, smooth muscle cells, keratinocytes, lymphocytes, and fibroblasts express IL-1α and IL-1β, as well. Released IL-1 acts on the T and B lymphocytes, macrophages, endothelium, and tissue cells inducing lymphocyte activation, macrophage stimulation, increased leukocyte/endothelial adhesion, fever due to hypothalamus stimulation, and release of acute-phase proteins by the liver (18).

#### 3.1.4 Interleukin-1α

Effects of IL-1α occur following the binding to DNA in the nucleus and binding to its cell membrane receptor and inducing downstream signaling. The translocation of the constitutive IL-1α between the cytosol and the nucleus results in distinct signaling. When the cell is under proapoptotic signaling, the cytosolic IL-1α travels to the nucleus and participates in transcription. Binding to the chromatin prevents inflammation. But under necrotic signals, the translocation of IL-1α to the cytosol occurs and a highly inflammatory response is the consequence (23). IL-1α, when released from necrotic cells, acts as a DAMP and its binding to the IL-1R1 receptor induces strong inflammation (24).

#### 3.1.5 Interleukin-1β

**Figure 1:**
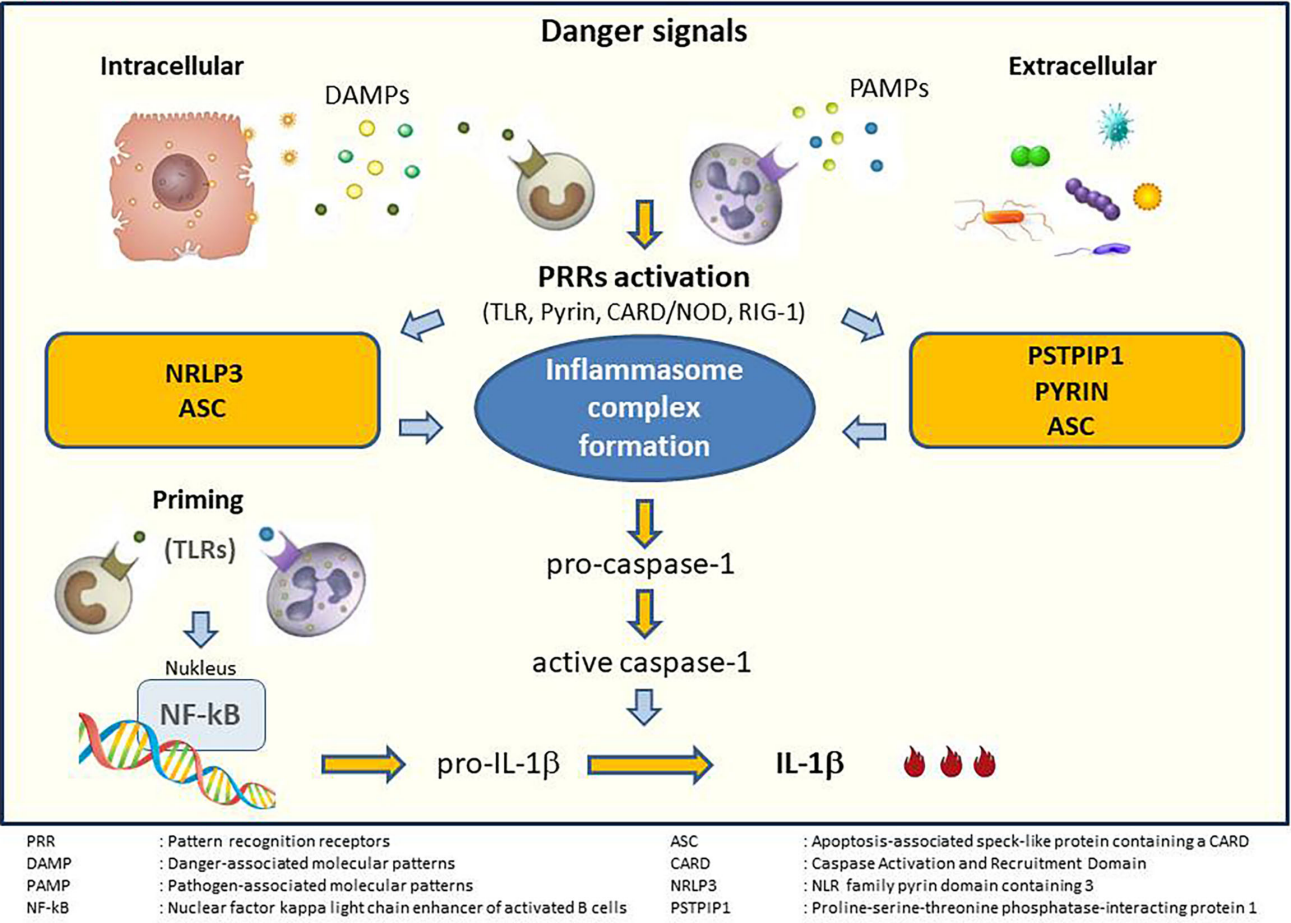
Generation of inflammasome and IL-1 activation. Adapted from Soy et al. (25).

Almost all microbial ligands use the Toll-like receptor (TLR) and induce the synthesis of IL-1β. This expression is induced by the transcription factor NF-κB. Upon stimulation, the innate immune cells release IL-1β as a precursor and its binding to the IL-1 receptor requires cleavage by a cysteine protease called caspase-1. Unlike IL-1α, IL-1β synthesis occurs only after stimulation. Pattern recognition receptors (PRRs) interact with apoptosis-associated speck-like protein containing a caspase-recruitment domain (ASC) and caspase-1. This interaction forms the inflammasome complex. This complex plays a crucial role in IL-1β maturation (**Figure 1**).

### 3.2 Functions of IL-1 in Health and Disease

**Figure 2:**
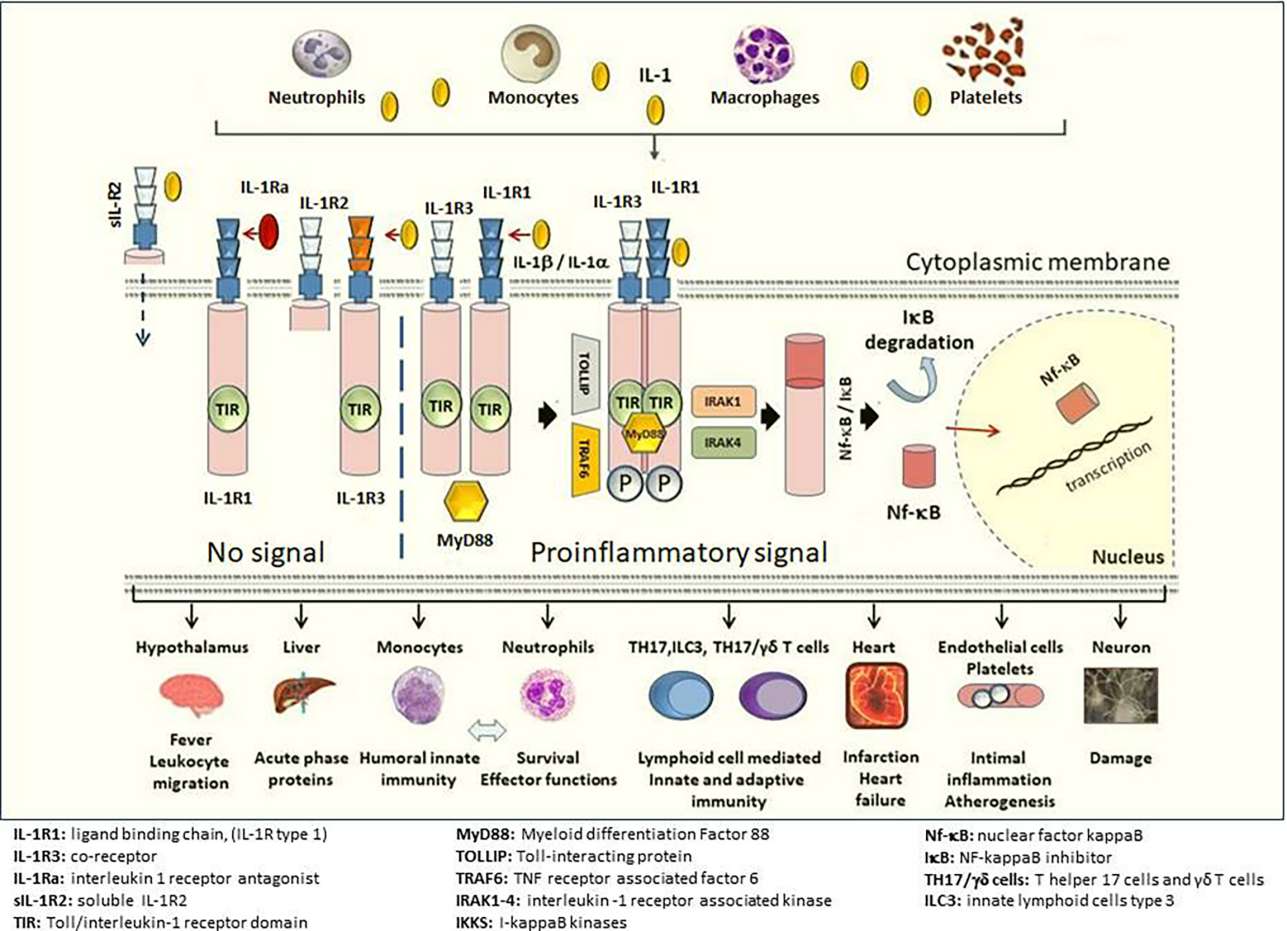
Downstream signaling of IL-1 and main target cells/tissues. The ligand-binding chain, IL-1R1 (IL-1R type 1), is present in all nucleated cells. The structural change that follows the binding of either IL-1β or IL-1α (not shown in the figure) to the IL-1R1 allows binding to co-receptor IL-1R3 (IL-1R type 3) and forms a heterotrimeric complex. The intracellular TIR domains of IL-1R1 and IL-1R3 bind MyD88. The phosphorylation of MyD88 and the participation of IL-1 receptor-activated kinases (IRAK 1-4) IkB degradation occur in the cytosol. NF-kB enters the nucleus and transcription induced by NF-kB results in a strong pro-inflammatory response. The binding of the IL-1Ra (IL-1R antagonist) to IL-1R1 does not cause a conformational change and IL-1R3 cannot bind, and hence, no signal transduction occurs despite presence of IL-1β. The affinity for IL-1Ra is weaker than for IL-1β, but stronger than that for IL-1α. IL-1β binds to IL-1R2 (IL-1R type 2) and together with IL-1R3 builds a trimeric complex. Since IL-1R2 lacks an intracellular-, as well as a TIR domain to dimerize with the TIR domain on IL-1R3, this trimeric complex does not create a signal. sIL-1R2 (soluble IL-1R2) in the extracellular space binds IL-1β and sequesters IL-1β away from IL-1R1 preventing the proinflammatory signal of IL-1β. Soluble IL-1R2 binds IL-1β and forms a complex with soluble IL-1R3 resulting in a higher affinity for IL-1β. Release of the proinflammatory cytokine IL-1β activates primarily innate immune cells such as neutrophils, inflammatory monocytes, as well as T helper 17 (TH17) cells, γδT cells, and innate lymphoid cells type 3 (ILC3). At the organ level, IL-1β is a key mediator of the fever response and pain processing in the hypothalamus. IL-1β promotes intimal inflammation and atherogenesis and increasing evidence suggests a pivotal role in the pathogenesis of thrombotic processes involved in myocardial infarction and acute heart failure thereafter. Illustration credit: Pamir Atagunduz. Similarly, it is suggested that IL-1 blocking may limit tissue damage after cerebral hemorrhage.

Activation of IL-1 results in several pro-inflammatory reactions but it is also constitutively expressed in many epithelial cells where it helps defending against pathogens and injury. More than 95% of known organisms depend on the cytokines of the IL1 superfamily for survival, whereas less than 5% depend on T- and B-cell functions (21, 26). In addition to the role IL-1 plays in innate immunity, IL-1 also regulates adaptive immune responses by inducing the differentiation of type 17 T-helper cells and IL-17 production. The contribution of IL-1 to local inflammation is mediated *via* an increase in levels of several enzymes such as phospholipase A2, COX, and eNos synthetase. Stimulation of neutrophil recruitment and degranulation by IL-1 enhances the inflammation and triggers the formation of NETosis. Furthermore, IL-1 promotes myelopoiesis, activates aggregation and adhesion of platelets that results in thrombosis (21, 26) (**Figure 2**).

#### 3.2.1 Disease States and IL-1

IL-1β seems to play an important role in the pathogenesis of various rheumatic diseases. Increased metalloproteinase activity, decreased proteoglycan production, and osteoclast activity induced by IL-1 may result in erosive changes in articular cartilage and the bones. First attempts to block IL-1 have failed to show an adequate response in rheumatoid arthritis (RA), SLE, and osteoarthritis (18, 27), but IL-1 blockade has proven to be effective in the treatment of autoinflammatory diseases (21). Many molecules are involved in the IL-1β activation cascade (**Figure 1**). Monogenic or polygenic genetic alterations in these molecules may lead to various diseases that are commonly referred to as autoinflammatory diseases. Monogenic autoinflammatory diseases include, but are not limited to Familial Mediterranean Fever (FMF), Tumor Necrosis Factor Receptor-Associated Periodic Syndrome (TRAPS), Cryopyrin-Associated Periodic Syndromes (CAPS), Hyperimmunoglobulinemia D syndrome/mevalonate kinase deficiency (HIDS/MKD), and deficiency of IL−1Ra (DIRA). Moreover, in the presence of permissive factors, IL-1 release may cause excessive immune cell recruitment and promotes overproduction of many cytokines, resulting in macrophage activation syndrome (MAS), and cytokine storm (25). Therefore, therapy against IL-1 may be effective in the treatment of these hyper-inflammatory conditions (28).

Increasing evidence suggests that treatment with IL-1 inhibition/neutralization will extend to diseases outside the rheumatologic scope and will include the treatment of more common diseases such as coronary heart diseases, i.e., myocardial infarction, and especially acute heart failure. When we searched clinicaltrials.gov with the keyword Interleukin-1 blockade, we found 10 studies. One of the them was about treatment of cardiac sarcoidosis, one about treatment of intracerebral hemorrhage by IL-1 blockers and others were about heart failure and treatment of IL-1 blocker (**Table 1**).

**Table 1:** Ongoing trials on IL-1 blockade*.

Title	Conditions	Interventions	Study Description
Interleukin-1 Blockade for Treatment of Cardiac Sarcoidosis (MAGiC-ART)	Cardiac Sarcoidosis	Usual Care Drugs: Anakinra Siltuximab Tocilizumab	In the current study, researchers aim to evaluate the safety and efficacy of IL-1 blockade with anakinra (IL-1 receptor antagonist) in patients with cardiac sarcoidosis.
BLOC-ICH: Interleukin-1 Receptor Antagonist in Intracerebral Haemorrhage (BLOC-ICH)	Intracerebral Haemorrhage	IL-1Ra: Kineret^®^ Placebo	In this study, researchers investigated the effects of Anakinra on intracerebral hemorrhage.
Interleukin-1 Blockade in HF With Preserved EF	Heart Failure With Normal Ejection Fraction	Drug: Anakinra Placebo	The main objective is to treat patients with HFpEF and evidence of systemic inflammation with an IL-1 blocker, anakinra or placebo to determine effects on exercise capacity measured as peak oxygen consumption at maximal cardiopulmonary exercise testing.
Interleukin-1 Blockade in Recently Decompensated Heart Failure (REDHART-1)	Heart Failure	Anakinra: (weeks 1-2) Anakinra: (weeks 3-12) Placebo	In this pilot study, researchers investigated the effects of two weeks use of the IL-1 receptor blocker Anakinra on Peak Oxygen Consumption, Quality of Life Improvement and Death or Hospital Admission for Heart Failure in patients with decompensated heart failure.
Interleukin-1 Blockade In Recently Decompensated Heart Failure - 2 (REDHART2)	Heart Failure, Systolic; Inflammation	Anakinra Placebo	Current study is a phase II clinical trial of anakinra or placebo to determine improvement in aerobic exercise capacity in patients with recently decompensated systolic heart failure (HF).
Interleukin-1 Blockade for the Treatment of Heart Failure in Patients With Advanced Chronic Kidney Disease	Heart Failure, Systolic Renal Disease, End Stage Chronic Kidney Diseases	Anakinra	The study was withdrawn because no patient enrollment could be made.
Interleukin-1 Blockade With Canakinumab to Improve Exercise Capacity in Patients With Chronic Systolic Heart Failure and Elevated High Sensitivity C-reactive Protein (Hs-CRP)	Prior Acute Myocardial Infarction Evidence of Systemic Inflammation (C Reactive Protein Plasma >2 mg/l) Reduced Left Ventricle Ejection Fraction (<50%) Symptoms of Heart Failure (NYHA Class II-III)	Canakinumab Cardiopulmonary exercise test Echocardiogram	In this trial researchers invastigate the effects of subcutaneous canakinumab in the prevention of recurrent cardiovascular events among stable post-myocardial infarction patients with elevated high sensitivity C-reaction protein.
Interleukin (IL)-1 Blockade in Acute Heart Failure (Anakinra ADHF)	Heart Failure	Anakinra (high dose), Anakinra (standard dose) Placebo	This study is a double-blind, randomized clinical trial investigating the effects of anakinra or placebo in patients with acute decompensated heart failure.
Interleukin-1 (IL-1) Blockade in Acute Myocardial Infarction (VCU-ART3)	Acute Myocardial Infarction	Anakinra 100 mg Placebo	This is a double-blind, randomized clinical trial of anakinra versus placebo in patients with ST-segment elevation myocardial infarction.
Fatigue and Interleukin -1 (IL-1) Blockade in Primary Sjøgrens Syndrome	Fatigue Primary Sjogren	Anakinra Placebo	In this study the investigators investigate the effects of Anakinra for fatique in patients with primary Sjogren’s Syndrome.

^*^ClinicalTrials.gov Search Results 09/17/2021.

Both the COVID-19 pandemic and vaccination against the SARS-CoV will continue to occupy the world agenda. The scarce data on this field and the expanding use of IL-1 blocking agents emphasize the need for further research of COVID-19 vaccination under these therapies. We did not find any ongoing studies on COVID-19 vaccines and IL-1 blockade, but we reached 10 studies with the keyword IL-1 and COVID19 at clincialtrail.gov website. These studies have generally investigated the effects of anakinra on hyperinflammatory state in the course of COVID-19.

### 3.3 Therapeutic Implications of IL-1 Inhibitors

Currently, three IL-1 targeted agents have been approved for the treatment of various auto-inflammatory diseases: Kineret, a recombinant interleukin (IL) 1 receptor antagonist (IL-1ra); canakinumab, a human monoclonal anti-IL-1β antibody, and rilonacept, a synthetic protein composed of the extracellular domains of IL-1R1 and interleukin-1 receptor accessory protein (IL-1RAcP) fused to a human IgG1 Fc domain. All three agents are administered by subcutaneous injection. In addition, a monoclonal antibody to the IL-1 receptor and a neutralizing anti-IL-1α antibody is under investigation in clinical trials (21). With a short half-life (4 to 6 h) anakinra is injected daily. Canakinumab has a longer half-life of 27 days and therefore it is injected every one to two months. Finally, rilonacept has 7 days half-life and is injected once weekly (28).

At present, three pharmacologic inhibitors of IL-1 are widely used for several indications.

#### 3.3.1 Anakinra and AIIRD

Anakinra (Kineret^®^), a recombinant, nonglycosylated form of the human interleukin-1 receptor antagonist (IL-1Ra), is currently approved for the treatment of RA, FMF, CAPS, DIRA, Still’s disease, such as Systemic Juvenile Idiopathic Arthritis (sJIA) and Adult-Onset Still’s Disease (AOSD), in adults, adolescents, children and infants aged 8 months and older with a bodyweight of 10 kg or above, in Europe and the US (29, 30).

#### 3.3.2 Canakinumab and AIIRD

Canakinumab (Ilaris^®^) is a neutralizing monoclonal antibody of IL-1β. It is currently approved for the treatment of periodic fever syndromes in adults, adolescents, and children older than 2 years, and the treatment of Still’s disease, sJIA, and gouty arthritis. Treatment with canakinumab is indicated for FMF, CAPS, TRAPS, and HIDS/MKD (31, 32).

#### 3.3.3 Rilonacept and AIIRD

Rilonacept (Arcalyst^®^), a soluble decoy receptor composed of the extracellular domains of IL-1R1 and IL-1RAcP fused to a human IgG1 Fc domain, is an interleukin-1 blocker indicated for the treatment of CAPS, namely, Familial Cold Autoinflammatory Syndrome (FCAS) and Muckle–Wells Syndrome (MWS) in adults and in children at least 12 years old. But as of September 2021, the drug has been voluntarily withdrawn from the market by the producing company for commercial reasons (33, 34).

IL-1 blockade has also been found to be successful in the treatment of hyperinflammatory state developing in the course of COVID-19 (27, 35).

## 4 Discussion

### 4.1 Vaccination and IL-1 Blockade

National authorities approved vaccination with twenty COVID-19 vaccines under emergency use authorizations, and six of these vaccines have been approved by the WHO, as well. As of September 14, 2021, over 5.6 billion vaccine doses have been administered worldwide (1). Interleukin (IL)-1 blockade has improved the outcome of autoinflammatory diseases (1). COVID-19 patients with AIIRD may be at higher risk of hospitalization, ICU admission, acute renal failure, and venous thromboembolism when compared to COVID-19 patients without systemic AIIRD (36). In addition, a recent study showed that patients with systemic rheumatic diseases and COVID-19 had an increased risk for hyperinflammation, kidney injury, admission to intensive care, and mechanical ventilation compared with matched controls (37).

At present, vaccination is the most effective method of preventing disease and death from infectious diseases, including COVID-19. It is essential to achieve the maximum benefit from vaccination, but its schedule should not compromise the state of therapy-induced low disease activity in immunosuppressed patients. Recent data on vaccination of patients with AIIRD treated with IL-1 blocking agents are summarized herein.

#### 4.1.2 IL-1 Blockade and Types of Vaccines in General

Compared to the general population, patients with AIIRD are more prone to influenza, pneumococci, human papillomavirus, and herpes zoster infections, and vaccination is effective in the prevention of these infectious diseases (38). Both, the American College of Rheumatology (ACR) and the European League Against Rheumatism (EULAR) recommendations strongly favor vaccination of patients with AIIRD (19, 39). These recommendations differ by type of vaccine.

#### 4.1.3 Live-Attenuated Vaccines

EULAR currently recommends that live-attenuated vaccines should be avoided in all age groups of patients with rheumatic diseases using IL-1 blocking agents (40–42). Recently, serious adverse events were reported in patients using IL-1 or IL-6 blocking agents after immunization with *live-attenuated* vaccines (43). In this retrospective-, multicenter study of 17 patients with sJIA and autoinflammatory diseases, varicella-zoster infection after varicella-zoster booster vaccination in one patient, and bacterial pneumonia after MMR booster in a second patient were observed (43). The clinically diagnosed varicella-zoster infection was treated with a 10-day course of intravenous acyclovir. This rare but critical report may support the EULAR recommendations. Disease flares were observed in seven patients, but in this retrospective study, the common approach observed among physicians was to stop treatment two to three days prior to and one or two days after vaccination with anakinra and about three months prior to and three months after the vaccination with canakinumab. Whether the interruption of treatment or the immunization was causative is hard to determine, but again disease flares after *live-attenuated* vaccines may be a further reason for avoidance.

#### 4.1.4 Inactivated Vaccines

The data on the safety and efficacy of *inactivated vaccines* in patients using IL-1 blocking agents is more comprehensive (44–47). Studies addressing vaccine immunogenicity using antibody titers after vaccination found that influenza vaccinations were effective in patients treated with canakinumab and anakinra (44, 46).

Likewise, Quartier et al. reported that the levels of postvaccination antibodies against the five pneumococcal capsular polysaccharide serotypes after one month were similar in patients under anakinra treatment with those treated with placebo (25, 46). Similarly, Camacho-Lovilla et al. have shown that biologic therapy with the anti-IL-1 receptor antagonist anakinra did not interfere with vaccine-induced protection in children with sJIA, and the majority had seroconversion to influenza A and B within 8 weeks remaining seropositive about a year after vaccination (48). Jaeger et al. assessed safety issues following influenza vaccines in adults treated with canakinumab for autoinflammatory diseases and reported no serious adverse events, but minor reactions in 7%. In addition, there were no disease flares (49). Therefore, immunization for influenza and meningococcal infections with inactivated vaccines may be recommended in all patients under treatment with all available anti-IL-1 agents.

The safety and efficiency of HPV vaccination under IL-1 blockade have not been studied well, but as an inactivated vaccine, immunization may be recommended for these patients, based on the long-term risks of HPV.

Recently, in a well-designed long-term study, antibody titers achieved after various non-live vaccines applied in childhood were addressed during treatment with canakinumab in a cohort of children with CAPS (45). Protective levels of antibodies against a broad spectrum of antigens after vaccination were detected in all 17 patients: *Corynebacterium diphtheriae*, *Bordetella pertussis*, *Neisseria meningitides*, *Clostridium tetani*, influenza A (H1N1 and H3N2), influenza B, Streptococcus pneumonia, and hepatitis B, diphtheria, pertussis, Meningococcus, tetanus, *Haemophilus influenza* B (polysaccharide or conjugate), and hepatitis A and B. In this extensive study, the mean exposure to canakinumab was 951 days per patient with a total of 44 patient-years, with the most common adverse event of nasopharyngitis in seven patients (41%).

However, the data on disease flares and adverse events after pneumococcal vaccination are conflicting. A recent study showed that CAPS patients treated with canakinumab reacted more frequently and more severely after pneumococcal vaccination in comparison to other non-live vaccines (49). In this study, 12 out of 18 patients who received pneumococcal immunizations developed vaccine reactions (fever, swelling, erythema, pain), usually within hours after vaccination. These reactions lasted up to three weeks. More importantly, pneumococcal vaccination triggered CAPS reactivation with systemic inflammation in two patients. Clinicians must balance the potential benefits of pneumococcal immunization against safety concerns. Probably the 13-valent pneumococcal conjugate vaccine should be chosen over the polysaccharide vaccine in CAPS patients.

### 4.2 IL-1 Blockade and COVID-19 Vaccines

Although reduced efficacy has been reported against new variants such as Delta, mRNA vaccines seem to be effective in 67 to 95% of cases in preventing symptomatic SARS-CoV-2 infection in those ≥16 years old (50). In the presence of limited data, it may be suggested that no modifications are needed for either immunomodulatory therapy or vaccination timing for those receiving anti-IL-1 treatment. But the degree of consensus on the timing of immunomodulatory therapy and vaccination under IL-1 blockade is still moderate (14, 15, 19).

The above recommendation for continuous immunomodulatory therapy with IL-1 blockers may be supported by the data of the case series and a meta-analysis of cohort studies addressing the use of IL-1 inhibitors in patients with COVID-19. There are also three observations supporting to continue IL-1 blockade in patients with AIIRD during COVID-19 vaccination: *i)* presence of very high IL-1 levels caused by disease activity itself, *ii)* additional elevation of IL-1 in patients with COVID-19 associated cytokine storm, and *iii)* better survival rates in patients with macrophage activation syndrome when treated with anakinra (51–53).

Similarly, registry data of children with multisystemic inflammatory syndrome show that neither the underlying autoinflammatory disease nor the ongoing treatment with IL-1 blockage predisposes to SARS-CoV-2 infection (54).

Further supporting evidence to the above suggestion of continuing anti-IL-1 therapy during COVID-19 vaccination might be the occurrence of flares in these patients. In a recent study of 17 children (seven with systemic juvenile idiopathic arthritis (sJIA), and ten with periodic fever syndromes) seven experienced flares of the underlying disease after COVID-19 vaccination, most probably due to discontinued therapy (43). But, as reflected by the moderate levels of agreement on the timing of immunomodulatory therapy and vaccination, some authors suggest that in patients treated with anti-cytokine therapies, vaccination for COVID-19 should be performed, if possible, seven days after the drug levels have returned to baseline (55).

#### 4.2.1 Seroconversion-Related Considerations

Both SARS-CoV-2 specific CD4^+^ and CD8^+^ T cells, as well as B cells against SARS-CoV-2 epitopes, have been found for up to 6 months after infection in about 95% of COVID-19 patients (56). This response may also be achieved with mRNA vaccines since they stimulate the humoral immune response directed to the SARS-CoV-2 spike protein (57–59). Additionally, mRNA vaccines can induce cellular immune responses of cytotoxic T lymphocytes (CTLs) that eliminate intracellular “latent” pathogens (60). There is no direct evidence that IL-1 blockade decreases antibody responses after COVID-19 vaccination. A recent, controlled small study in patients with chronic inflammatory diseases treated mostly with TNF inhibitors and anti-IL-17 agents reported that anti-SARSCoV-2-IgG levels achieved in these patients were slightly lower, but similar to controls. This observational study provides the first time data that SARS-CoV-2 mRNA vaccines efficiently lead to the development of antibodies in immunosuppressed patients (61). Additionally, it has been previously shown that a single dose of 300 mg canakinumab s.c. does not affect the induction or persistence of antibody responses after vaccination with unadjuvanted influenza or alum-adjuvanted MenC vaccines in healthy subjects (44). All these findings might support the recommendation that patients under IL-1 blockade may be vaccinated for COVID-19 without interrupting the treatment (14, 15, 19).

## 5 Conclusion

Only a small number of studies address vaccination of AIIRD patients under immunosuppressive treatment with IL-1 blocking agents such as canakinumab, anakinra, or rilonacept. The scarce data available on the efficiency of vaccines under general immunosuppression for conventional vaccines arises many questions concerning SARS-CoV-2 vaccination. Until the emergence of further data, physicians should adhere to general recommendations for patients with autoimmune inflammatory diseases (41, 42, 62). Blockade of IL-1 is vital in autoinflammatory diseases and these patients require long-term or lifelong immunosuppression to prevent specific organ damage. COVID-19 has a more pronounced course in these patients with uncontrolled disease. Therefore, individual patient and disease-associated characteristics and the actual need for IL-1 blockade should be incorporated into the decision-making process.

Following recommendations may guide physicians:

1. Patients with rheumatic diseases, including autoimmune and autoinflammatory diseases, should receive the COVID-19 vaccine.
2. Scientific evidence is still limited for COVID-19 immunization under immunosuppression.
3. Patients should be incorporated into the decision-making.
4. Non-live vaccines are generally safe and provide effective immunity under IL-1 blockade except for pneumococcal vaccination, which causes minor adverse events including fever and more importantly, frequent disease flares. In these patients, the 13-valent pneumococcal conjugate vaccine may be chosen rather than the polysaccharide vaccine.
5. COVID-19 vaccines, namely, the mRNA vaccines are not live vaccines and can be administered to immunocompromised patients.
6. The efficiency of the COVID-19 vaccine may be expected to be lower in patients treated with IL-1 blockade, but limited evidence shows that patients with chronic rheumatic diseases under immunosuppression with either anti-TNF agents or IL-17 blockade develop adequate levels of antibody responses.
7. With a level of moderate agreement, it may be recommended that patients under IL-1 blockade can be vaccinated without interrupting the anti-cytokine therapy, especially in patients with ongoing high disease activity to avoid disease relapses.
8. In selected cases, after balancing for disease activity and risk of relapses, vaccination may be given seven days after the drug levels have returned to baseline, especially for IL-1 blocking agents with long half-lives such as canakinumab and rilonacept. This may help to ensure an ideal vaccine response in the face of the possibility that AIIRD patients may develop a more pronounced and severe COVID-19 disease course.

## Author Contributions

This article was designed and written by PA, GK, and MS. The figures were drawn by PA. All authors contributed to the article and approved the submitted version.

## Conflict of Interest

The authors declare that the research was conducted in the absence of any commercial or financial relationships that could be construed as a potential conflict of interest.

## Publisher’s Note

All claims expressed in this article are solely those of the authors and do not necessarily represent those of their affiliated organizations, or those of the publisher, the editors and the reviewers. Any product that may be evaluated in this article, or claim that may be made by its manufacturer, is not guaranteed or endorsed by the publisher.
